# Comparative evaluation of deep learning architectures, including UNet, TransUNet, and MIST, for left atrium segmentation in cardiac computed tomography of congenital heart diseases

**DOI:** 10.12771/emj.2025.00087

**Published:** 2025-04-21

**Authors:** Seoyeong Yun, Jooyoung Choi

**Affiliations:** Ewha Womans University College of Medicine, Seoul, Korea

**Keywords:** Cardiovascular diseases, Congenital heart defects, Deep learning, Heart atria, Precision medicine

## Abstract

**Purpose:**

This study compares 3 deep learning models (UNet, TransUNet, and MIST) for left atrium (LA) segmentation of cardiac computed tomography (CT) images from patients with congenital heart disease (CHD). It investigates how architectural variations in the MIST model, such as spatial squeeze-and-excitation attention, impact Dice score and HD95.

**Methods:**

We analyzed 108 publicly available, de-identified CT volumes from the ImageCHD dataset. Volumes underwent resampling, intensity normalization, and data augmentation. UNet, TransUNet, and MIST models were trained using 80% of 97 cases, with the remaining 20% employed for validation. Eleven cases were reserved for testing. Performance was evaluated using the Dice score (measuring overlap accuracy) and HD95 (reflecting boundary accuracy). Statistical comparisons were performed via one-way repeated measures analysis of variance.

**Results:**

MIST achieved the highest mean Dice score (0.74; 95% confidence interval, 0.67–0.81), significantly outperforming TransUNet (0.53; P<0.001) and UNet (0.49; P<0.001). Regarding HD95, TransUNet (9.09 mm) and MIST (5.77 mm) similarly outperformed UNet (27.49 mm; P<0.0001). In ablation experiments, the inclusion of spatial attention did not further enhance the MIST model’s performance, suggesting redundancy with existing attention mechanisms. However, the integration of multi-scale features and refined skip connections consistently improved segmentation accuracy and boundary delineation.

**Conclusion:**

MIST demonstrated superior LA segmentation, highlighting the benefits of its integrated multi-scale features and optimized architecture. Nevertheless, its computational overhead complicates practical clinical deployment. Our findings underscore the value of advanced hybrid models in cardiac imaging, providing improved reliability for CHD evaluation. Future studies should balance segmentation accuracy with feasible clinical implementation.

## Introduction

### Background

Accurate segmentation of cardiac structures in chest computed tomography (CT) images is critical for the diagnosis, treatment planning, and management of cardiovascular diseases. Segmentation enables detailed visualization of cardiac anatomy, facilitating the identification of congenital defects, structural anomalies, and pathological changes. Precise segmentation also provides essential information for surgical preparation and interventional procedures, improving surgical outcomes and patient safety. Furthermore, quantitative analyses such as ventricular volume measurements, ejection fraction calculations, and assessments of wall motion are crucial for evaluating cardiac function and diagnosing conditions like heart failure.

Deep learning has revolutionized medical image segmentation, with architectures such as UNet becoming foundational due to their capacity to capture global context and fine-grained details. UNet, introduced by Ronneberger et al. [1] in 2015, follows an encoder-decoder structure with skip connections, yielding remarkable performance across diverse biomedical imaging tasks. However, UNet has limitations in modeling complex spatial relationships and long-range dependencies. To address these shortcomings, advanced models such as TransUNet and MIST have been developed. TransUNet incorporates transformer modules to effectively capture global contextual information [2], while MIST employs multi-scale feature integration strategies and attention mechanisms to further refine segmentation [3]. Collectively, these models represent an evolution from traditional convolutional neural networks (CNNs) toward state-of-the-art hybrid approaches, emphasizing integrated local and global feature representations to increase segmentation accuracy. These 3 models are detailed in Supplement 1.

Despite advancements in deep learning-based segmentation, few studies have comprehensively evaluated state-of-the-art models specifically for cardiac chamber segmentation of chest CT images. Accurate delineation of the left atrium (LA) provides valuable insights into chamber volumes, wall thickness, and morphological abnormalities, which greatly inform early diagnosis and clinical decision-making. However, manual segmentation remains labor-intensive and susceptible to inter-observer variability, underscoring the need for robust and automated segmentation solutions.

Although individual studies have separately investigated UNet, TransUNet, or MIST for cardiac segmentation, a direct comparison of their performance on a common dataset has not yet been reported. While numerous reports have addressed whole-heart or multi-chamber segmentation, this research specifically focuses on the LA, addressing the gap in the literature by evaluating these models to clarify their strengths and limitations. The selection of UNet, TransUNet, and MIST reflects the progressive evolution of segmentation model design.

### Objective

The primary objective of this study is to compare the effectiveness of 3 deep learning models—UNet, TransUNet, and MIST— for segmentation of the LA in cardiac CT images of patients with congenital heart diseases (CHDs). Specifically, the study aims to evaluate segmentation performance using the Dice score and HD95 metrics. Additionally, various architectural modifications within the MIST model, including multi-scale attention, skip connections, and hybrid encoders, are investigated to understand their impacts on segmentation accuracy.

## Methods

### Ethics statement

This study was exempt from institutional review board approval because it used only publicly available, de-identified data. Specifically, we utilized the ImageCHD dataset [4], which contains no personally identifiable information and is licensed under the Apache License 2.0. The dataset was obtained from Kaggle (https://www.kaggle.com/) and used under its open-access terms.

### Study design

This is a prediction study involving a comparative analysis of the performance of deep learning models for LA segmentation in cardiac CT images. It follows the TRIPOD+AI reporting guidelines for studies of deep learning models in medical applications (development or prediction), available at: https://www.tripod-statement.org/.

### Setting

The ImageCHD dataset comprises 3-dimensional (3D) CT images acquired using a Siemens Biograph 64 scanner (Siemens Healthineers) from 110 patients with CHDs, aged from 1 month to 40 years, predominantly between 1 month and 2 years. The dataset documentation did not specify recruitment dates or the participating institution.

### Participants

Participant-specific information was not provided, as all data were de-identified.

### Data sources

This study employed the ImageCHD dataset, sourced from Kaggle, for evaluating the UNet, TransUNet, and MIST models. The dataset includes 110 high-quality 3D CT scans annotated with segmentation labels for cardiac structures such as the LA, left ventricle, right ventricle, right atrium, myocardium, aorta, and pulmonary arteries. Designed to facilitate segmentation of major heart structures critical for CHD classification, it contains volumetric scans with LA labels to support the assessment of segmentation accuracy. As an open-source resource, this dataset is dedicated to individuals diagnosed with CHD and emphasizes cardiac structures, particularly the LA. Its annotations facilitate rigorous evaluation and comparative analysis of segmentation models, thereby advancing automated diagnostics in CHD imaging. Of the 110 CT images provided, 108 were included in this study due to quality concerns with 2 images.

### Outcome variables

Segmentation performance was quantified using the Dice score (reflecting overall overlap) and HD95 (reflecting boundary accuracy).

### Study size

All available cases in the dataset meeting the quality criteria were extracted and utilized; thus, no formal sample size estimation was performed.

### Data preprocessing

Data preprocessing was performed to ensure consistency and improve model performance. Two-dimensional (2D) samples were extracted by slicing the 3D H×W×D arrays along the depth axis (D), yielding a series of 2D H×W images for subsequent analysis. The 2D samples were normalized to a standard intensity range (for all models) and augmented to improve model generalizability. The 2D slices were normalized to a standard intensity range (for all models) and augmented to improve model generalizability. Data augmentation included random rotations (±10°), translations (up to 5% width/height shifts), and zooming (±10%) for UNet, applying nearest-neighbor interpolation for image data and constant filling (value of 0) for segmentation masks.

### Deep learning models

#### Experimental setup

All models (UNet, TransUNet, and MIST) were trained on image volumes resized to 512×512 pixels. Experiments were conducted on Google Colab (Google LLC) using NVIDIA GPUs (L4 for UNet and TransUNet, T4 and L4 for TransUNet, and Tesla V100 for MIST; NVIDIA Corp.) and implemented with PyTorch (https://pytorch.org/). TensorBoard (https://www.tensorflow.org/) was employed to monitor training progress, visualizing losses, and performance metrics in real time across epochs.

#### Model configurations

For UNet, a weighted focal categorical cross-entropy loss function was utilized with an Adam optimizer and a learning rate of 1×10^-5^. For TransUNet, the loss function combined categorical cross-entropy and Dice coefficient loss; the optimizer was stochastic gradient descent with an initial learning rate of 0.001, subsequently reduced by a factor of 0.1 during training.

**Fig. 1.**
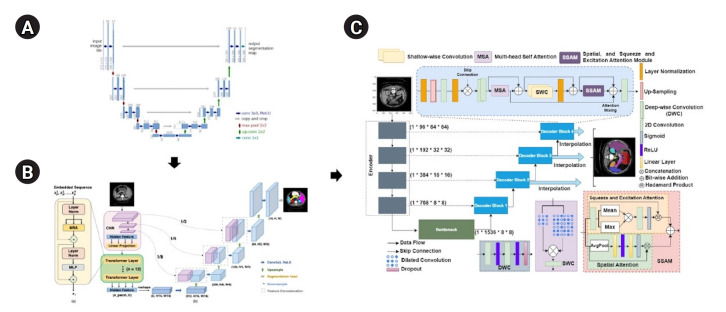
Model architectures. (A) UNet (Ronneberger et al. [1], 2015), (B) TransUNet (Chen et al. [2], 2021), and (C) MIST (Rahman et al. [3], 2023). A brief overview explains the evolution of transformer and UNet hybrid models. The UNet architecture introduced symmetric encoder-decoder paths with skip connections. TransUNet is built upon UNet by adding transformer attention mechanisms to the encoder, and MIST, a recent model, further integrates multiple attention mechanisms and optimized skip connections to improve performance.

The MIST model employed a combined Dice loss and binary cross-entropy loss to manage class imbalance, optimized using Adam with an initial learning rate of 0.001 and similarly reduced by a factor of 0.1 throughout training. Additionally, the MIST decoder was tested with and without the spatial squeeze-and-excitation attention module (SSAM), employing attention-based or concatenation strategies, as depicted in Fig. 1. Output aggregation strategies included summing feature maps from decoder blocks or exclusively utilizing the final feature map (Fig. 1).

### Training and evaluation

All 3 models (UNet, TransUNet, and MIST) were trained and evaluated using the ImageCHD dataset. Of the 108 cases, 97 were partitioned randomly into training (approximately 80%, 77 cases) and validation (approximately 20%, 20 cases) sets. The remaining 11 cases served as an independent test set for all models. Consistent data splits were maintained across models to ensure fairness in performance comparisons. For evaluation, slices were preprocessed to 512×512 pixels and normalized uniformly.

### Performance metrics

Segmentation performance was evaluated using the Dice score and HD95 metrics. The Dice score quantifies the overlap between predicted segmentation and ground truth labels, providing a value between 0 (no overlap) and 1 (perfect overlap). It is mathematically defined as follows:


(1) $$DICE\left(X,Y\right)=\frac{2*\left|X\cap Y\right|}{\left|X\right|+\left|Y\right|}$$

Where X represents the set of predicted pixels and Y the set of ground truth pixels.

The HD95 metric measures the distance between the boundaries of the predicted and true segmentations. It calculates the 95th percentile of the Hausdorff distance to reduce sensitivity to outliers, thus providing a robust measure of segmentation boundary accuracy. HD95 was calculated on 2D slices without applying in-plane voxel spacing (0.25 mm), and thus the reported values are in pixel units rather than millimeters. The out-of-plane spacing (0.5 mm) was not relevant since only axial 2D slices were used.


(2) $$HD95=max\left(d_{X}\;,\;d_{Y}\right)$$

The software and tools utilized included Python 3.8 (https://www.python.org/), deep learning frameworks PyTorch 1.8 and TensorFlow, and supplementary libraries including NumPy, SciPy, scikit-learn, and OpenCV for image processing. The source code for this study is provided in Supplement 2.

### Statistical methods

The metrics (Dice score and HD95) were statistically compared using one-way repeated measures analysis of variance for paired samples. DBSTAT 5 for Windows (DBSTAT Co.) was used for statistical analysis.

## Results

### Participants/dataset

The ImageCHD dataset is an open-source collection of cardiac CT images available on Kaggle, specifically focused on patients with CHDs (Dataset 1).

#### Model performance

**Fig. 2.**
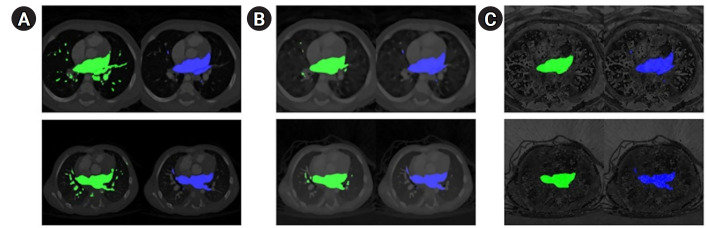
Qualitative segmentation outcomes across model architectures. The ground truth (blue) is presented in the second column, while the predicted segmentation (green) is shown in the left column. (A) UNet, (B) TransUNet, and (C) MIST. Detailed results, including dice scores and 95th percentile of the Hausdorff distance (HD95) values for individual computed tomography scans, are provided in Supplements 3 and 4.

Detailed loss performance data are available in Dataset 2. Example comparisons of qualitative segmentation outcomes across different model architectures are illustrated in Fig. 2.

**Fig. 3.**
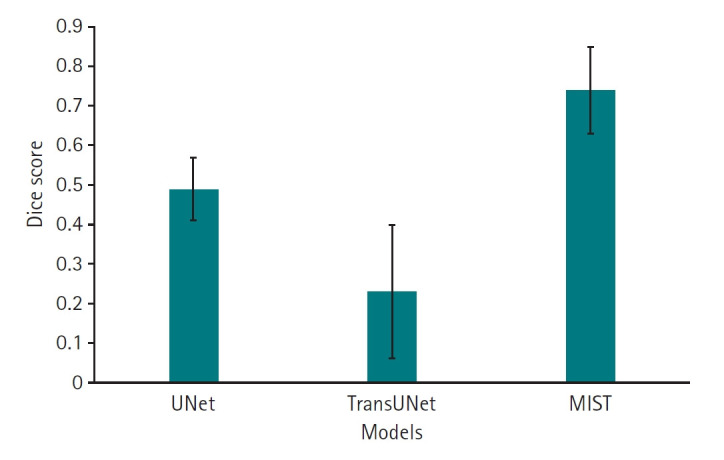
Dice scores for 11 left atrial cardiac computed tomography images segmented by UNet, TransUNet, and MIST models.

**Fig. 4.**
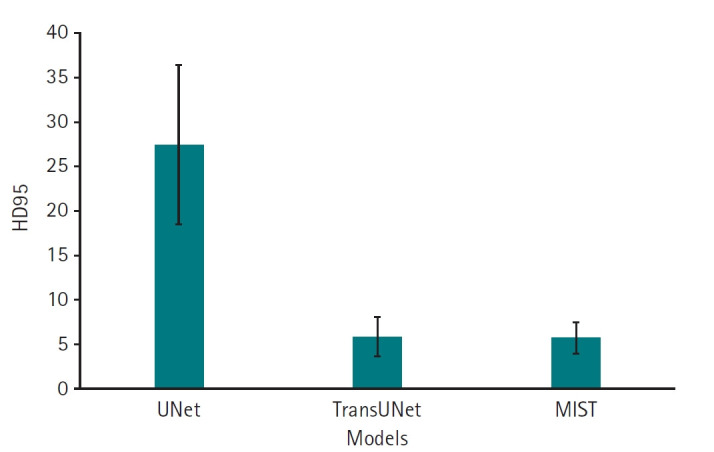
95th percentile of the Hausdorff distance (HD95) values for 11 left atrial cardiac computed tomography images segmented by UNet, TransUNet, and MIST models.

Dice scores for the 3 deep learning models are presented in Fig. 3. The results, including Bonferroni’s multiple comparison, demonstrate that MIST achieved the highest performance, with a mean Dice score of 0.74 (95% confidence interval [CI], 0.67–0.81), significantly outperforming the other 2 models (P<0.001). UNet (mean, 0.49; 95% CI, 0.43–0.55) outperforemed TransUNet (mean, 0.23; 95% CI, 0.12–0.34) (P<0.001) (Supplement 3). Regarding HD95, which measures boundary accuracy, both TransUNet (mean, 5.85 mm; 95% CI, 4.32–7.38 mm) and MIST (mean, 5.77 mm; 95% CI, 4.55–6.98 mm) significantly outperformed UNet (mean, 27.49 mm; 95% CI, 21.45–33.53 mm) (P<0.001). The difference in HD95 performance between TransUNet and MIST was not statistically significant (P=1.0000) (Fig. 4, Supplement 4).

**Fig. 5.**
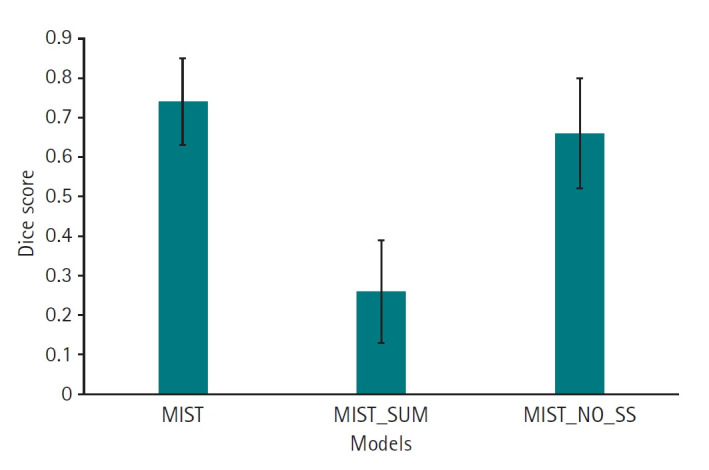
Dice scores for 11 left atrial cardiac computed tomography images segmented by MIST with or without spatial squeeze-and-excitation attention module (SSAM) or sum output heads.

**Fig. 6.**
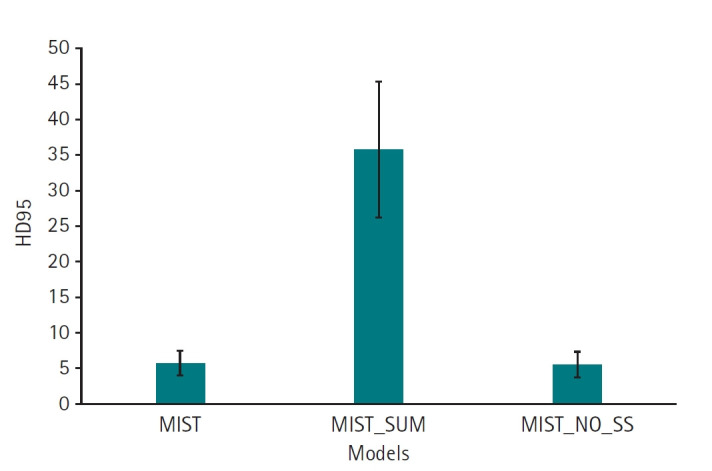
95th percentile of the Hausdorff distance (HD95) values for 11 left atrial cardiac computed tomography images segmented by MIST with or without spatial squeeze-and-excitation attention module (SSAM) or sum output heads.

**Fig. 7.**
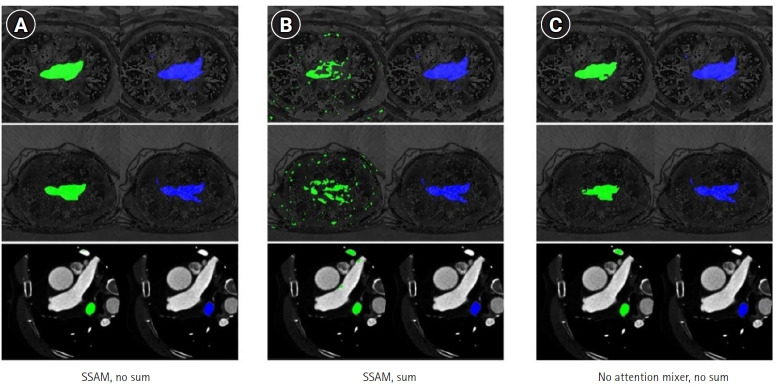
Qualitative outcomes for MIST variants. The ground truth (blue) is presented in the second column, while the predicted segmentation (green) is shown in the left column. Starting from left: spatial squeeze-and-excitation attention module (SSAM) without summation (A), SSAM with summation (B), and no attention mixer within the decoder and no summation (C).

Results from ablation experiments, in which individual parameters were varied systematically, are presented in Figs. 5 (Supplement 5) and 6 (Supplement 6), with measured results available in Dataset 3. The MIST model, incorporating SSAM within the decoder blocks and without sum output heads, exhibited a higher Dice score (mean, 0.74; 95% CI, 0.67–0.81) compared to variations employing sum output heads (mean, 0.26; 95% CI, 0.17–0.35). However, the performance of the MIST model without SSAM (mean, 0.66; 95% CI, 0.57–0.76) was comparable to that of MIST with SSAM (Fig. 5, Supplement 5). For HD95, the MIST variant with SSAM and without sum output heads also demonstrated superior performance (mean, 5.77 mm; 95% CI, 4.55–6.98 mm) relative to versions employing sum output heads (mean, 35.88 mm; 95% CI, 29.43–42.32 mm). The HD95 of MIST without SSAM (mean, 5.59 mm; 95% CI, 4.35–6.82 mm) was comparable to the MIST model with SSAM and without sum output heads (Fig. 6, Supplement 6). Example images in Fig. 7 illustrate how the best-performing model accurately delineated the atrial borders and exhibited fewer false predictions in distant anatomical regions.

## Discussion

### Key results

MIST achieved the highest mean Dice score (0.74; 95% CI, 0.67–0.81), significantly outperforming TransUNet (0.23) and UNet (0.49). TransUNet and MIST also outperformed UNet in HD95, with no significant difference. Parameter variations revealed that incorporating SSAM into MIST while omitting sum output heads yielded the highest Dice score and the lowest HD95 value.

### Interpretation

These results suggest that MIST’s optimized skip connections and multi-scale attention mechanisms are beneficial for capturing the subtle anatomical variations involved in LA segmentation in children with CHD. Although TransUNet achieved HD95 performance comparable to MIST, the Dice score is generally regarded as the primary indicator of segmentation quality, since it averages performance across the entire region and is less sensitive to localized outliers. In comparison, HD95—while valuable for assessing boundary precision, especially when accurate delineation is critical—can be more sensitive to outliers. Consequently, unless an application explicitly demands extremely precise boundary localization (e.g., surgical planning), overall overlap as captured by the Dice score is typically prioritized. We therefore recommend MIST as the preferred overall segmentation method for LA structures in CHD imaging.

The failure of SSAM to improve LA segmentation performance likely arises from several factors, including the nature of the target anatomy [5], the design of the attention module [6], and the existing capabilities of the MIST model. Accurate segmentation of the LA, a small structure with subtle boundaries, depends on capturing fine-grained features that generic spatial attention mechanisms may overlook.

### Comparison with previous studies

Although numerous investigations have applied attention mechanisms or transformer‐based architectures to medical image segmentation, a direct comparison of UNet, TransUNet, and MIST on a single, consistent dataset for LA segmentation in cardiac CT images of patients with CHD remains absent. To our knowledge, no prior research has comprehensively benchmarked these 3 models head-to-head under uniform experimental conditions, making this study novel in its direct evaluation.

We therefore must contextualize our findings against earlier studies that employed attention-based or transformer-based architectures for similar segmentation tasks, despite differences in target anatomy or datasets. Our findings align with existing research indicating that CNN-based models such as UNet struggle to capture long-range dependencies due to their fixed receptive fields [1]. Transformer-based architectures like TransUNet address this limitation by integrating global context [2], although they frequently face challenges in preserving spatial precision, particularly for small or irregular structures [7,8].

Hybrid models such as MIST, which combine multi-scale attention with refined skip connections, have demonstrated superior segmentation performance [3]. Our results further support recent findings suggesting that such attention-driven hybrid architectures outperform both CNN-only and transformer-only models [9].

Unlike prior studies focusing on multi-chamber heart segmentation, our work provides a dedicated evaluation of LA segmentation, offering key insights into which architectural elements most benefit this task.

### Strengths

Unlike prior studies that segment multiple chambers or the entire heart, this work targets LA segmentation, enabling a detailed and clinically relevant analysis. Beyond overall performance, we dissect key architectural components, particularly the SSAM and the multi-head attention mechanisms in MIST, thus contributing to future advancements. Specifically, we performed additional ablation experiments to clarify their contributions and inform the model optimization process.

### Limitations

This study had several limitations. First, the overall dataset was relatively small, potentially limiting model generalizability. Expanding analysis to larger and more diverse datasets will be crucial for further validation.

Second, the data may have introduced bias due to limitations on scanner diversity and representation of patient populations.

Third, although we used L4 GPUs to ensure consistent hardware conditions, performance and runtime metrics may differ on other GPU architectures. Future studies should normalize batch sizes, standardize training epochs, and benchmark across uniform hardware environments to control for computational heterogeneity.

Finally, some methodological limitations should be noted. Potential biases in study design and dataset selection may have influenced our results, despite efforts to maintain evaluation consistency. Moreover, we did not apply probability calibration to segmentation outputs. Future work should explore calibration techniques such as Platt scaling or isotonic regression to improve confidence estimation and support safer clinical adoption.

### Clinical implications

With its high segmentation accuracy, MIST can assist radiologists in detecting subtle LA abnormalities and improve risk assessment for atrial fibrillation. As a pre-screening tool, MIST automates initial LA segmentation and reduces radiologists’ workload during final review. We recommend integrating MIST into hospital picture archiving and communication systems (PACS), thus enabling streamlined deployment and improving accessibility in real-world clinical settings.

### Suggestion for further studies

To advance the clinical utility and robustness of deep learning-based cardiac segmentation, future research should prioritize the following directions:

First, optimize the MIST framework to reduce computational and memory requirements while preserving segmentation accuracy, enabling real-time clinical deployment.

Second, extend segmentation tasks to other anatomical regions (such as the brain, lungs, and additional cardiovascular structures) to evaluate broader applicability. Conduct multicenter validation using data from diverse clinical settings to improve generalizability across populations and imaging protocols.

Third, develop techniques to increase transparency and foster trust among clinicians, such as attention maps and saliency analysis.

Fourth, explore semi-supervised or unsupervised learning approaches to minimize reliance on large labeled datasets, lowering barriers to adoption in resource-constrained environments.

Fifth, investigate scalable deployment strategies, including integration with PACS and cloud-based platforms, to streamline workflows and facilitate clinical use.

### Conclusion

MIST demonstrated superior dice score performance compared with UNet and TransUNet, while also achieving improved boundary accuracy (lower HD95) relative to UNet. These promising results are primarily attributable to its integration of multi-scale attention mechanisms and optimized skip connections. Validation confirmed the model’s robustness across various conditions, although its higher computational complexity poses challenges for real-time clinical deployment without further optimization. Overall, this study advances automated cardiac segmentation by incorporating hybrid attention mechanisms, providing insights for future model development.

## Footnotes

**Data availability**
Files are available from Harvard Dataverse: https://doi.org/10.7910/DVN/VGUF3Z Dataset 1. The ImageCHD dataset used in this study is publicly available on Kaggle under the Apache 2.0 License: https://www.kaggle.com/datasets/xiaoweixumedicalai/imagechd. Dataset 2. Dice score and HD95 values for each cardiac CT sample across MIST, UNet, and TransUNet. HD95, 95th percentile of the Hausdorff distance; CT, computed tomography. Dataset 3. Dice score and HD95 values obtained by MIST with or without SSAM and with or without sum output heads. HD95, 95th percentile of the Hausdorff distance; SSAM, spatial squeeze-and-excitation attention module.

## Supplementary materials

Supplementary files are available from Harvard Dataverse: https://doi.org/10.7910/DVN/4EWPMF

Supplement 1. Detailed explanations of the UNet, TransUNet, and MIST models.

Supplement 2. Full source code for UNet, TransUNet, and MIST, including training scripts, model architectures, and evaluation procedures, which is also available at https://github.com/janet-rgb/CardiacSegmentation.

Supplement 3. One-way repeated measures analysis of variance (paired sample) for dice scores across the 3 models.

Supplement 4. One-way repeated measures analysis of variance (paired sample) for HD95 values across the 3 models.

Supplement 5. One-way repeated measures analysis of variance (paired sample) for dice scores of MIST variations (with/without SSAM and with/without sum output heads).

Supplement 6. One-way repeated measures analysis of variance (paired sample) for HD95 values across MIST variations (with/without SSAM and with/without sum output heads).
