# Supplementary material for: Comparative evaluation of deep learning architectures, including UNet, TransUNet, and MIST, for left atrium segmentation in cardiac computed tomography of congenital heart diseases
Source: Ewha Med J. 2025 Apr 21;48(2):e33. doi: 10.12771/emj.2025.00087 (PMC12277500; doi:10.12771/emj.2025.00087)
Supplement: Supplementary file 1 — Supplement 1. Detailed explanations of the UNet, TransUNet, and MIST models. [file emj-2025-00087-Supplement-1.docx]

Supplement 1. Detailed explanations of deep learning models, including U-Net, TransUNet, and MIST.

The U-Net architecture is characterized by its unique symmetric design, consisting of a contracting encoder and an expansive decoder connected via skip connections. The encoder employs multiple convolutional layers with ReLU activations and max pooling to progressively extract spatially hierarchical features, while the decoder uses transposed convolutions to upsample these features and reconstruct fine-grained spatial details. Skip connections directly concatenate feature maps from corresponding encoder layers to decoder layers, preserving high-resolution information lost during downsampling. This structure enables U-Net to segment complex medical images with high precision. However, the reliance on fixed kernel sizes and convolutional operations may limit its ability to capture large-scale contextual information, and the skip connections can inadvertently introduce noise from irrelevant high-frequency details, necessitating careful preprocessing and optimization.

  TransUNet integrates the strengths of convolutional neural networks (CNNs) and transformers into a unified architecture tailored for medical image segmentation. The model begins with a CNN-based encoder, derived from U-Net, to extract low-level spatial features through convolution and pooling operations. These features are then flattened and fed into a transformer module, which employs multi-head self-attention to model long-range dependencies and global contextual relationships. The transformer output is reshaped and passed to a decoder that mirrors the U-Net design, utilizing skip connections to combine high-resolution features from the encoder with globally informed representations from the transformer. This architecture not only enhances the segmentation of complex anatomical structures by capturing fine details and global context simultaneously but also effectively mitigates U-Net's limitations in handling large-scale dependencies within medical images.

  TransUNet enhances the traditional U-Net by integrating a Transformer encoder to capture long-range dependencies and global context, addressing the limitations of CNNs in modeling complex structures. The model combines CNN-based local feature extraction (from U-Net) with the global attention mechanism of Transformers, ensuring both fine-grained details and global context are preserved. It is achieved by using a CNN backbone for low-level feature extraction and a Transformer encoder. The decoder integrates features from both the CNN and Transformer, retaining U-Net's skip connections. This hybrid architecture enhances the model's ability to understand both local and global features.

  The MIST model advances beyond TransUNet by introducing a novel Convolutional Attention Mixing (CAM) decoder, which integrates Multi-head Self-Attention, Spatial Attention, and Squeeze Attention for more effective feature refinement and decoding. Unlike TransUNet, which primarily relies on a Transformer encoder for global context, MIST incorporates MaxViT blocks [5] in the encoder, combining grid attention and block attention for improved multi-scale feature learning. Additionally, the CAM decoder, coupled with U-Net-style skip connections, enhances both segmentation accuracy and structural similarity by synergizing local and global attention mechanisms more effectively than TransUNet’s architecture. This comprehensive attention-driven approach allows MIST to capture complex spatial and structural dependencies with greater precision. [3]
